# The relationship between nuclear factor (NF)-κB family gene expression and prognosis in triple-negative breast cancer (TNBC) patients receiving adjuvant doxorubicin treatment

**DOI:** 10.1038/srep31804

**Published:** 2016-08-22

**Authors:** Ji-Yeon Kim, Hae Hyun Jung, Soomin Ahn, SooYoun Bae, Se Kyung Lee, Seok Won Kim, Jeong Eon Lee, Seok Jin Nam, Jin Seok Ahn, Young-Hyuck Im, Yeon Hee Park

**Affiliations:** 1 Division of Hematology-Oncology, Department of Medicine, Samsung Medical Center, Sungkyunkwan University Seoul 06351, Korea; 2 Department of Health Sciences and Technology, SAIHST, Sungkyunkwan University, Seoul 06351, Korea; 3 Innovative Cancer Medicine Institute, Samsung Medical Center, Sungkyunkwan University Seoul 06351, Korea; 4 Department of Surgery, Samsung Medical Center, Seoul 06351, Korea; 5 Biomedical Research Institute, Samsung Medical Center, Sungkyunkwan University, Seoul 06351, Korea.

## Abstract

We investigated gene expression profiles of the NF-κB pathway in patients with triple-negative breast cancer (TNBC) receiving adjuvant chemotherapy to determine the prognostic value of NF-κB pathway genes according to chemotherapeutic regimen. We used the nCounter expression assay to measure expression of 11 genes (NFKB1, NFKB2, RELA, RELB, REL, TP53, FOXC1, TBP, SP1, STAT3 and IRF1 genes) belonging to the NF-κB pathway using mRNA extracted from paraffin-embedded tumor tissues from 203 patients diagnosed with TNBC. Of the 203 patients, 116 were treated with a chemotherapeutic regimen containing doxorubicin. As revealed by the expression profiles of the 11 genes, increased expression of SP1 was associated with poor prognosis in TNBC patients treated with adjuvant doxorubicin chemotherapy (5-year distant recurrence-free survival [5Y DRFS], low vs. high expression [cut-off: median]: 92.3% vs. 71.6%, *P* = 0.001). In a multivariate Cox regression model, SP1 expression was a useful marker for predicting long-term prognosis in TNBC patients receiving doxorubicin treatment, and we thus suggest that SP1 expression could serve as a prognostic marker in these patients.

Breast cancer is the most common cancer in women worldwide^1^. Of breast cancers, triple-negative breast cancer (TNBC), which is defined by the absence of hormone receptor expression and HER2 overexpression, has more aggressive biologic features and poor prognosis^2^,^3^,^4^. In addition, TNBC has heterogeneous histologic characteristics^5^ and is associated with complex genetic alterations^6^. However, in spite of therapeutic advances in the potential druggable genetic alterations of TNBC, such as MARK and PARP inhibitors and anti-PD-L1 antibody, no target molecules have been identified for TNBC. Accordingly, cytotoxic chemotherapy remains the most effective treatment strategy for TNBC.

Doxorubicin, a cytotoxic agent affiliated with anthracycline, is the most active agent used to treat breast cancer in neoadjuvant, adjuvant and palliative settings^7^,^8^,^9^. In the adjuvant setting, doxorubicin-containing chemotherapy significantly reduced breast cancer recurrence and mortality^7^,^10^,^11^. No targeted agents are available for TNBC in an adjuvant setting and thus conventional cytotoxic chemotherapy is particularly important.

Doxorubicin inhibits DNA and RNA synthesis by intercalation between DNA/RNA strand pairs^12^,^13^ and inhibits topoisomerase II enzyme, thus blocking DNA transcription and replication^14^. However, the DNA damage induced by doxorubicin activates the NF-κB pathway, leading to doxorubicin resistance in cancer cell lines, including in breast cancer cells^15^,^16^,^17^,^18^.

The transcription factor NF-κB family consists of five gene members: RELA (RelA, p65), RELB (RelB), REL (c-Rel), NFKB1 (NF-κB1, p105) and NFKB2 (NF-κB2, p100). NF-κB transcription factors bind as dimers to κB sites in enhancers and promoters of various genes to regulate transcription^19^,^20^, and are also tightly controlled at multiple regulatory element levels^20^,^21^. SP1 activates RELA^22^ and REL transcription^23^, and STAT3 is a transcription factor for RELA, RELB and NFKB2^24^. Additional transcription factors include TBP and FOXC1 for NFKB1, TP53 for NFKB2 and IRF1 for REL transcription^25^. Moreover, a recent study showed that p53 depletion is required for NF-κB target gene activation by doxorubicin chemotherapy^26^.

These five family gene members control two main NF-κB pathways: the canonical pathway composed by RelA and the non-canonical pathway associated with RelB and NF-κB2^20^. In breast cancer cells, the canonical pathway of NF-κB is associated with doxorubicin resistance^26^,^27^,^28^. Moreover, studies using human breast cancer tissue have shown that high nuclear RelA expression promotes activation of the canonical NF-κB pathway and is associated with poor prognosis^26^,^29^,^30^. However, these studies were conducted regardless of breast cancer molecular subtypes based on the presence or absence of hormone receptor expression or HER2 overexpression. Moreover, there has been no previous study on the NF-κB pathway and doxorubicin resistance in TNBC.

Previous reports suggested that methotrexate inhibits NF-κB activity^31^. Tumor cells with an inactivated NF-κB pathway are sensitive to 5-fluorouracil chemotherapy^32^, while a gastric cancer cell line with NF-κB pathway activation acquired 5-fluorouracil chemotherapy resistance^33^.

We conducted this study to reveal how transcription factor NF-κB family genes and their regulatory elements affect response to adjuvant chemotherapy, including doxorubicin, in patients with TNBC.

## Results

### Patient characteristics

A total of 203 TNBC patients who underwent curative surgery and adjuvant chemotherapy were enrolled (Supplementary Fig. 1 and Table 1). One patient was excluded due to missing chemotherapy regimen records. Of the remaining 202 patients, 116 were treated with doxorubicin-containing chemotherapy and 86 received cyclophosphamide, methotrexate and 5-fluorouracil (CMF) chemotherapy. Of the 116 patients, 58 (50.0%) were treated with doxorubicin, cyclophosphamide and 5-fluorouracil (FAC) chemotherapy, 17 (14.7%) received doxorubicin and cyclophosphamide (AC) and 41 (35.3%) received AC followed by taxane (AC-T) chemotherapy.

The selection of chemotherapy regimen depended on diagnostic stage (*P* < 0.001) (Table 2). Most patients with stage I/IIA breast cancer were treated with CMF or FAC chemotherapy (61.8% and 29.1% in stage I and 48.9% and 34.0% in stage IIA, respectively), whereas patients with stage IIB/IIIA and IIIC breast cancer received AC-T chemotherapy (48.5% in stage IIB, 69.2% in stage IIIA and 87.5% in stage IIIC).

The expression profile of NF-κB pathway-associated genes is described in Supplementary Table 1 and Supplementary Fig. 2. For further survival analysis, we set the median expression score of the 11 genes as the cut-off value to divide patients into two groups based on lower and higher expression.

### NF-κB Pathway gene expression profile

Interactions among genes expressed in the NF-κB pathway are described in Supplementary Fig. 3 and we performed Pearson correlation analysis to explore associations among the expressed genes. The eleven NF-κB pathway-associated genes were classified into two categories: one consisted of NFKB1, NFKB2, REL, RELA and RELB (NF-κB pathway genes) and the other consisted of SP1, STAT3, TBP, FOXC1, IRF1 and TP53 (regulatory genes of NF-κB pathway genes).

Among the NF-κB pathway genes, NFKB1 expression was positively correlated with REL expression (Pearson R: 0.694, *P* < 0.001), and NFKB2 was positively correlated with RELA and RELB expression (Pearson R between NFKB2 and RELA: 0.825, *P* < 0.001 and Pearson R between NFKB2 and RELB: 0.728, *P* < 0.001; Supplementary Table 1).

Analysis of the association between the six regulatory genes and five NF-κB pathway genes revealed that higher SP1 or STAT3 expression was strongly correlated with RELA overexpression (Pearson R: 0.845, *P* < 0.001 of SP1 and 0.697, *P* < 0.001 of STAT3).

### Impact of baseline characteristics, including NF-κB pathway gene expression, on distant recurrence-free survival according to chemotherapy regimen

**Figure 1:**
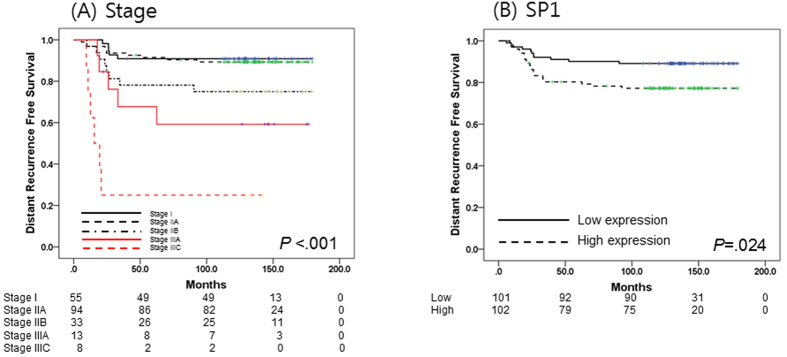
Survival analysis in patients with triple negative breast cancer (TNBC) (N = 203). (**A**) Kaplan-Meier survival curve of stage at diagnosis. (**B**) Kaplan-Meier survival curve of SP1 expression.

Of 203 patients, 34 patients (16.7%) experienced breast cancer recurrence. Median follow-up duration was 137 months. In univariate analysis, the five-year DRFS (5Y DRFS) rate in patients with stage I and IIA disease was 90.9% and 91.5%, respectively, compared to 78.8%, 67.7%, and 25.0% in patients with stage IIB, IIIA, and IIIC disease, respectively (*P* < 0.001) (Table 1 and Fig. 1A). Chemotherapy regimen affected DRFS (*P* = 0.001), but was also highly related to disease stage (*P* < 0.001) (Tables 1 and 2).

Of NF-κB pathway-associated genes, the level of SP1 gene expression influenced DRFS in TNBC patients (5Y DRFS, low vs. high: 91.1% vs. 80.3%, *P* = 0.024) (Table 1 and Fig. 1B). In addition, the level of NFKB1 and RELA expression also marginally impacted DRFS (5Y DRFS, [low vs. high expression of NFKB1: 81.2% vs. 89.1%, *P* = 0.057], [low vs. high expression of RELA: 90.1% vs. 80.3%, *P* = 0.061]).

**Figure 2:**
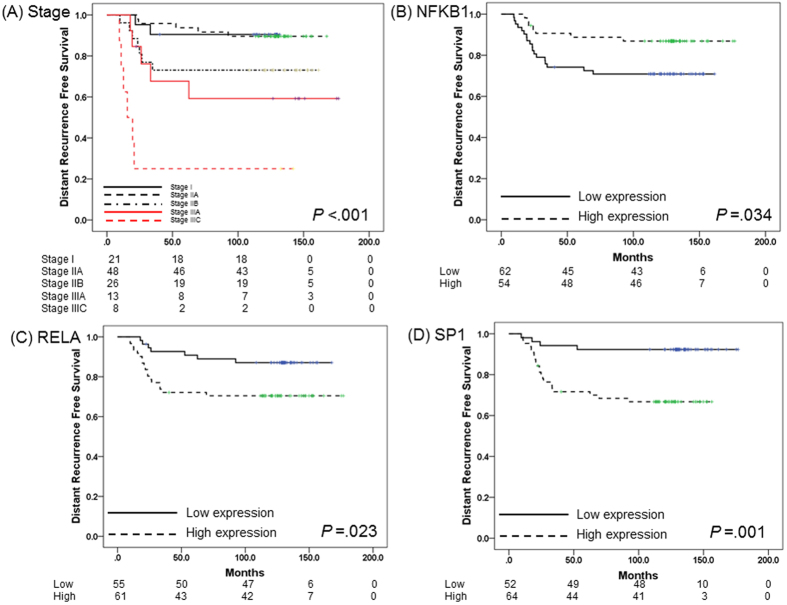
Survival analysis in TNBC treated with adjuvant doxorubicin chemotherapy (N = 116). (**A**) Kaplan-Meier survival curve of stage at diagnosis. (**B**) Kaplan-Meier survival curve of NFKB1 expression. (**C**) Kaplan-Meier survival curve of RELA expression. (**D**) Kaplan-Meier survival curve of SP1 expression.

In subgroup analysis of patients who received doxorubicin chemotherapy, disease stage significantly affected DRFS. 5Y DRFS rates for stage I and IIA disease were 90.5% and 93.8%, respectively, in contrast with 73.1%, 67.7% and 25.0% for stage IIB, IIIA and IIIC, respectively (*P* < 0.001) (Table 2 and Fig. 2A). With regard to gene expression profile, low expression of NFKB1 and high expression of RELA and SP1 were associated with short DRFS duration ([5Y DRFS for NFKB1, low vs. high: 74.2% vs. 88.8%, *P* = 0.034], [5Y DRFS for RELA, low vs. high: 90.8% vs. 72.1%, *P* = 0.023], [5Y DRFS for SP1, low vs. high: 92.3% vs. 71.6%, *P* = 0.001]) (Table 1 and Fig. 2B–D).

Furthermore, we performed multivariate analysis of factors associated with patient prognosis in univariate analysis, including stage, chemotherapeutic agent and NFKB1, RELA and SP1 gene expression in patients with doxorubicin chemotherapy. Stage and expression level of SP1 remained statistically significant factors affecting DRFS ([hazard ratio (HR) of DRFS 1.34 (95% confidence interval [CI] 0.26–6.95) for stage IIA, 3.60 (95% CI 0.75–17.36) for stage IIB, 7.07 (95% CI 1.36–36.91) for stage IIIA, and 20.19 (95% CI 4.00–101.87) for IIIC, *P* < 0.001], [HR of DRFS 4.97 (95% CI 1.68–14.73) for high SP1 expression, *P* = 0.004]) (Table 3).

**Figure 3:**
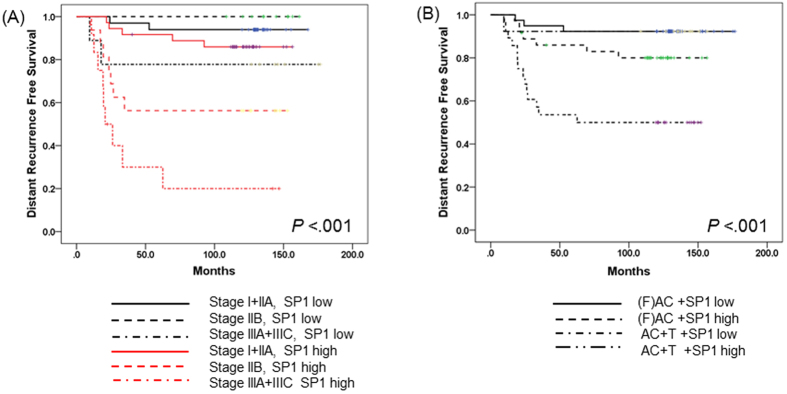
(**A**) Survival analysis according to stage and SP1 expression. (**B**) Survival analysis according to chemotherapy and SP1 expression.

The effect of SP1 expression on DRFS was more intensive in advanced-stage TNBC patients treated with adjuvant doxorubicin chemotherapy. For stage IIIA and IIIC, the 5Y DRFS rate in patients with high SP1 expression was 30.0%, compared to 77.8% in patients with low SP1 expression. Patients who overexpressed SP1 with stage I/IIA and IIB TNBC had 5Y DRFS of 91.7% and 56.3%, respectively, while those with low expression of SP1 had 5Y DRFS of 93.9% and 100.0%, respectively (*P* < 0.001) (Fig. 3A). In addition, we analyzed the co-effect of taxane chemotherapy and SP1 expression on survival. In this analysis, regardless of additional docetaxel treatment, high SP1 expression was significantly associated with DRFS ([5Y DRFS in high SP1 expression, (F)AC and AC+T chemotherapy: 85.9% and 53.6%], [low SP1 expression: 92.3% and 92.3%], *P* < 0.001) (Fig. 3B). We examined the impact of baseline characteristics on DRFS in patients who received adjuvant CMF chemotherapy, but SP1 expression had no significant effect on DRFS (Table 2).

**Figure 4:**
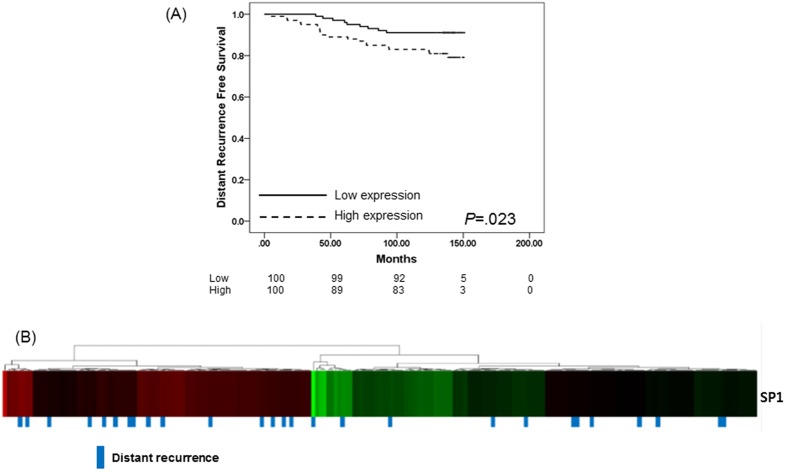
Survival analysis in the validation cohort (N = 202). (**A**) Kaplan-Meier curve of distant recurrence-free survival according to SP1 expression. (**B**) Heat map for SP1 mRNA expression and distant recurrence status.

Lastly, we validated the impact of SP1 expression on breast cancer disease progression. Using breast cancer tissue from 202 patients diagnosed with breast cancer since 2003 to 2004, we tested the level of SP1 mRNA expression and analyzed the effect of SP1 expression level on DRFS. Analysis indicated that high SP1 mRNA expression was associated with distant recurrence of breast cancer (*P* = 0.023) (Fig. 4A,B). Median follow-up duration was 139.8 months and 29 patients (14.4%) had distant recurrence of cancer; 20 patients (19.8%) with high SP1 expression had tumor recurrence in contrast to only nine patients (8.9%) with low SP1 expression.

### Predictive value of SP1 expression in TNBC patients treated with doxorubicin

**Figure 5:**
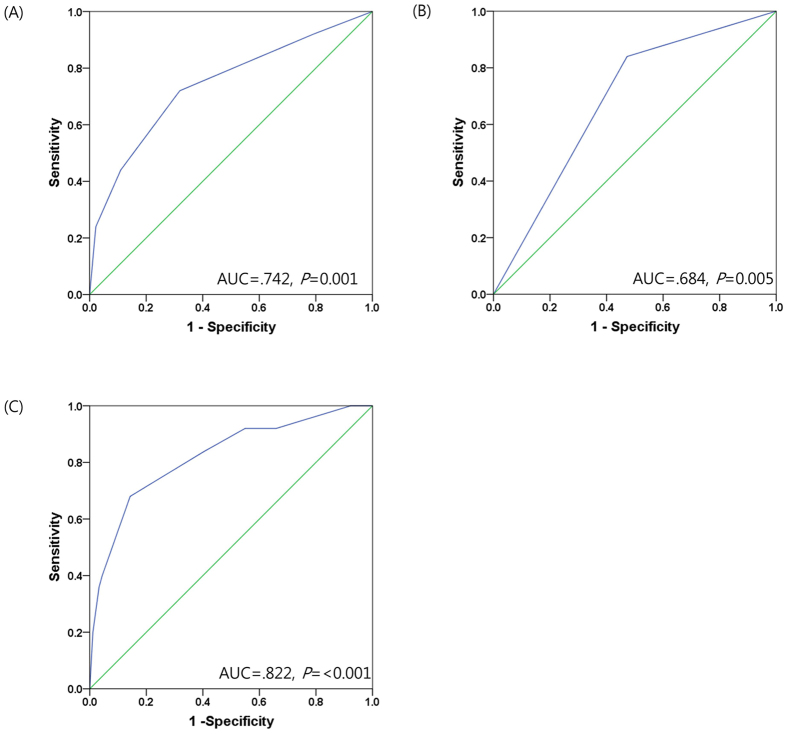
ROC analysis of the predictive accuracy of stage and SP1 expression for distant recurrence-free survival of patients with TNBC receiving adjuvant doxorubicin chemotherapy (N = 116). (**A**) ROC curve of stage. (**B**) ROC curve of SP1 expression. (**C**) ROC curve of stage and SP1 expression.

Receiver operating characteristics(ROC) analysis was performed to evaluate the predictive value of SP1 expression level. Adding SP1 expression level to TNM stage enabled prediction of DRFS in patients who received adjuvant doxorubicin chemotherapy. The results of ROC analysis revealed that SP1 expression strengthened the predictive efficacy of TNM stage ([TNM stage: AUC: 0.742, *P* = 0.001], [SP1 expression: AUC: 0.684, *P* = 0.005] and [TNM stage + SP1 expression: AUC: 0.822, *P* < 0.001]) (Fig. 5).

## Discussion

In this study, we demonstrated the impact of NF-κB pathway gene expression on the prognosis of TNBC and found that SP1 gene expression level was a potential prognostic marker in TNBC patients receiving adjuvant doxorubicin chemotherapy.

**Figure 6:**
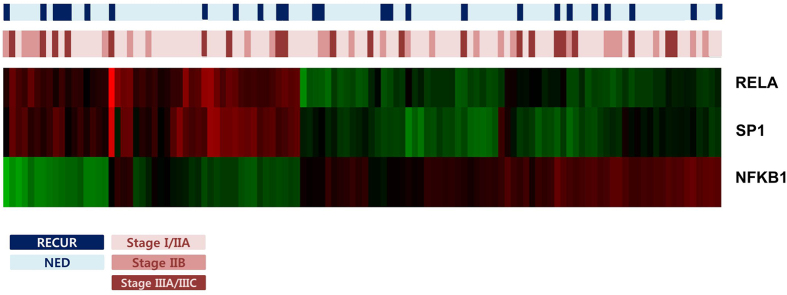
Heatmap for SP1, RELA and NFKB1 expression.

Many previous studies have shown that RelA expression is a predictive marker for doxorubicin chemotherapy^26^,^29^,^30^ by examining intra-nuclear staining using RelA antibody and detecting protein expression using intra-nuclear RelA expression score. In our study, we performed the nCounter expression assay to measure the expression of eleven genes: five canonical and non-canonical NF-κB pathway genes and six associated regulatory factors. With this approach, we sensitively detected gene expression regardless of intra-nuclear or extra-nuclear expression. In our results, univariate analysis revealed that three canonical NF-κB pathway genes, SP1, RELA and NFKB1^34^, were related to doxorubicin resistance (Fig. 6).

Of these three genes, only SP1 maintained statistical significance in multivariate analysis, although SP1 and RELA expression were strongly associated; patients with high expression of SP1 also exhibited high RELA expression.

Sp1, part of the Sp/KLF family of transcription factors, is a zinc finger transcription factor that binds to GC-rich motifs of many promoters^35^. Several genes, including RelA, are regulated by Sp1 and high Sp1 expression promotes cell growth, cell survival and gene expression, causing carcinogenesis. Sp1 overexpression is a negative prognostic marker in pancreatic cancer and gastric cancer^36^,^37^ and Sp1 downregulation inhibits cell survival of rhabdomyosarcoma^38^. In addition, Sp1 overexpression induces doxorubicin resistance in HL-60, a myeloid leukemia cell line^39^.

Sp1 is a downstream target of many pathways in addition to NF-kB, including MAPK, and JNK pathways. SP1 and the other 10 measured genes are also involved in many genetic pathways. However, we only evaluated Sp1 and NF-kB pathway genes and did not perform pathway activity analysis. Therefore, our suggestion that SP1 and NFkB activation induced anthracycline resistance had limitation.

However, many previous studies already showed that canonical NF-kB pathway activation was positively correlated to anthracycline resistance using pathway analysis. Additionally, RELA and SP1 expression patterns interacted with each other and high SP1 and RELA expression indicated a high recurrence rate of TNBC. This result was similar to that of previous studies on the relationship between anthracycline resistance and canonic NF-kB pathway activation. Accordingly, we might suggest that the level of SP1 expression indicated canonic NF-kB pathway activity and thus is a potential prognostic marker of TNBC treated with anthracycline.

Multiple myeloma is a well-known hematologic malignancy that is regulated by Sp1 transactivation and the NF-κB pathway, and downregulation of Sp1 and RelA induces tumor regression^40^. Moreover, bortezomib, a gangbuster drug for multiple myeloma^41^, is a potent, highly selective, and reversible proteasome inhibitor that targets the 26S proteasome complex and inhibits its function. Besides inhibiting NF-κB, bortezomib inhibited tumor cell growth by targeting cell cycle regulatory proteins, the unfolded protein response (UPR) pathway, p53-mediated apoptosis, and DNA repair mechanisms as well as classical stress response pathways. Bortezomib suppresses Sp1 activity and disrupts the physical interaction of Sp1/RelA, ultimately downregulating the NF-kB pathway^42^. Accordingly, bortezomib represents a potential therapeutic strategy for anthracycline-resistant TNBC by activating Sp1 and NF- kB genes. Although a previous phase I/II study of bortezomib and capecitabine in patients with metastatic breast cancer found a moderate antitumor effect in heavily pretreated patients^43^, breast cancer patients with an activated NF-κB pathway might benefit from bortezomib treatment.

TP53 is a tumor suppressor gene; some reports have indicated that silencing of p53 expression is associated with anthracycline resistance in breast cancer^26^,^44^. However, we did not find any relationship between TP53 expression and prognosis. In addition, TP53 expression did not affect the expression of NF-κB pathway genes.

Our study is the first to demonstrate the impact of NF-κB pathway gene expression in TNBC patients treated with adjuvant doxorubicin chemotherapy. Our findings suggest that Sp1 expression is positively correlated with RelA expression. Moreover, high expression of Sp1 is associated with poor prognosis in patients with TNBC treated with adjuvant anthracycline chemotherapy. Considering the heterogeneous molecular characteristics of TNBC, Sp1 is not only a potential predictor of anthracycline response, but also a classification factor for clarifying the heterogeneity of TNBC. Furthermore, accurate prediction of anthracycline chemotherapy outcomes could change current practice guideline of adjuvant chemotherapy in TNBC^45^. Moreover, bortezomib, a proteasome inhibitor, is a potential therapeutic strategy for anthracycline-resistant TNBC through its activation of Sp1 and NF- kB genes.

## Methods

### Patients

This study was conducted via retrospective analysis of the clinical records of patients with invasive breast cancer who received adjuvant chemotherapy after curative surgery at Samsung Medical Center between 2000 and 2004. Women diagnosed with stage I to IIIC breast cancer were included. At initial diagnosis, medical history, physical examination, blood tests, mammography, breast ultrasonography, breast magnetic resonance imaging (MRI), abdominal computed tomography (CT) scan, and bone scans and/or positron emission tomography (PET)-CT scans (if indicated) were performed (Fig. 1).

The institutional review board of Samsung Medical Center, Seoul, Korea approved our study protocol and waived the need for informed consent due to the use of archival tissue samples and retrospective clinical data (IRB No: 2012-08-065).

### RNA extraction

We examined hematoxylin and eosin (H&E)-stained slides from all available archival formalin-fixed, paraffin-embedded (FFPE) primary breast tumor tissue. Two independent pathologists reviewed all pathology specimens to determine the following tumor characteristics: histological grade^46^, nuclear grade, ER/progesterone receptor (PgR) expression and HER2 overexpression.

RNA was extracted from two to four 4-μm-thick FFPE sections that contained tumor tissue using the High Pure RNA Paraffin kit (Roche Diagnostic, Mannheim, Germany). RNA yield and purity were assessed using a NanoDrop ND-1000 Spectrophotometer (NanoDrop Technologies, Rockland, DE, USA). Samples with a total RNA concentration less than 50 ng/uL were excluded from analysis, because 200 ng of input RNA in 5 ul was used for hybridization with 20 uL of probe set master mix.

### nCounter expression assay (NanoString)

The NanoString nCounter Analysis System (NanoString Technologies, Seattle, WA, USA) was used to measure the amount of gene expression. Using a multiplexed hybridization assay and digital readouts of fluorescent probes^47^, this system measures the relative abundance of each mRNA transcript. We used an nCounter CodeSet (NanoString Technologies) containing biotinylated capture probes for NFKB1, NFKB2, RELA, RELB, REL, TP53, FOXC1, TBP, SP1, STAT3 and IRF1 genes and 5 housekeeping genes and reporter probes attached to color barcode tags according to the nCounter code-set design. The CodeSet was hybridized in solution to 200 ng of total RNA for 18 h at 65 °C according to the manufacturer’s instructions.

Hybridized samples were loaded into the nCounter Prep Station for post hybridization processing. On the deck of the Prep Station, hybridized samples were purified and immobilized in a sample cartridge for data collection, followed by quantification of target mRNA in each sample using the nCounter Digital Analyzer. Quantified expression data were analyzed using NanoString’s nSolver Analysis Software.

After performing image quality control using a predefined cutoff value, we excluded outlier samples using a normalization factor based on the sum of positive control counts greater than threefold. Counts of the probes were then normalized using the geometric mean of the five housekeeping genes and log2 transformed for further analysis.

### Statistical analysis

DRFS was defined as the elapsed time from the date of curative surgery to the detection of disease recurrence. DRFS was analyzed using the Kaplan-Meier method. Univariate and multivariate analyses of DRFS were performed using Cox’s proportional hazards regression tests. To evaluate the relationship among expression of the eleven genes, we use Pearson correlation analysis. Lastly, ROC analysis was performed to evaluate the prognostic value of the expression level of NF-kB family genes and ligands adding weight. ROC analysis was conducted by adding the weighted value of gene expression on a pre-existing known prognostic marker already validated using univariate analysis. Two-tailed P values of <0.05 were considered statistically significant and IBM SPSS Statistics 21 for Windows (IBM Corp., Armonk, NY, USA) was used to analyze all data.

### Remark guidelines

We have adhered to the guidelines of a methodological paper from 2005 entitled “Reporting recommendations for tumor marker prognostic studies (REMARK guidelines)”^48^,^49^. To minimize any potential bias arising from review of medical records, we included “Patient Cohort” analysis to fulfill these criteria (Supplementary Figure 1).

## Additional Information

**How to cite this article**: Kim, J.-Y. *et al.* The relationship between nuclear factor (NF)-κB family gene expression and prognosis in triple-negative breast cancer (TNBC) patients receiving adjuvant doxorubicin treatment. *Sci. Rep.* **6**, 31804; doi: 10.1038/srep31804 (2016).

## Supplementary Material

Supplementary Information

## Figures and Tables

**Table 1:** Impact of baseline characteristics on patient prognosis (N = 203).

	Total N = 203 (%)	5Y DRFS (%)	*P*-value
Age (median)	46.4 ± 10.2		
Range	23.5–74.1		0.633
<40 YO^1^	48 (23.6)	81.2	
≥40 YO	155 (76.4)	86.4	
Histology			0.507
IDC^2^	180 (88.7)	85.5	
Other	23 (11.3)	82.9	
Stage			<0.001
I	55 (27.1)	90.9	
IIA	94 (46.3)	91.5	
IIB	33 (16.3)	78.8	
IIIA	13 (6.4)	67.7	
IIIB	0 (0)		
IIIC	8 (3.9)	25.0	
Nuclear grade			0.258
1	2 (1.0)	50.0	
2	47 (23.2)	82.8	
3	145 (71.4)	86.9	
Unknown	9 (4.4)	77.8	
Histologic grade			0.704
1	3 (1.5)	100.0	
2	45 (22.2)	84.4	
3	144 (70.9)	86.0	
Unknown	11 (5.4)	72.7	
Adjuvant chemotherapy			0.001
CMF^3^	86 (42.4)	90.7	
FAC^4^	58 (28.6)	86.0	
AC^5^	17 (8.4)	100.0	
AC –T^6^	41 (20.2)	65.9	
Unknown	1 (0.5)	100.0	
Adjuvant RTx^5^			0.093
Yes	130 (64.0)	83.0	
No	73 (36.0)	89.0	
NFKB1 (median: 25.60)			0.057
Low	101(49.9%)	81.2	
High	102(50.1%)	89.1	
NFKB2 (median: 297.30)			0.793
Low	101(49.9%)	85.1	
High	102(50.1%)	85.3	
RELA (median: 224.85)			0.061
Low	101(49.9%)	90.0	
High	102(50.1%)	80.4	
RELB (median: 61.25)			0.567
Low	101(49.9%)	86.1	
High	102(50.1%)	84.3	
REL (median: 65.48)			0.411
Low	101(49.9%)	82.2	
High	102(50.1%)	88.2	
TP53 (median: 221.06)			0.957
Low	101(49.9%)	85.3	
High	102(50.1%)	85.1	
FOXC1 (median: 126.94)			0.678
Low	101(49.9%)	87.1	
High	102(50.1%)	83.3	
TBP (median: 92.68)			0.307
Low	101(49.9%)	87.1	
High	102(50.1%)	83.3	
SP1 (median: 100.01)			0.024
Low	101(49.9%)	90.1	
High	102(50.1%)	80.3	
STAT3 (median:1599.68)			0.732
Low	101(49.9%)	86.1	
High	102(50.1%)	84.3	
IRF1 (median: 192.75)			0.758
Low	101(49.9%)	86.1	
High	102(50.1%)	84.3	

^1^Years old, ^2^Invasive ductal carcinoma, ^3^Cyclophospamide/Methotrexate/Fluorouracil, ^4^Fluorouracil/Adriamycin/Cyclophosphamide, ^5^Adriamycin/Cyclophosphamide, ^6^Taxane, ^7^Radiotherapy.

**Table 2:** Impact of baseline characteristics on patient prognosis according to adjuvant chemotherapy (N = 202).

	Doxorubicin N = 116 (%)	5Y DRFS (%)	*P*-value	CMF^1^ N = 86 (%)	5Y DRFS (%)	*P*-value
Age (median)	46.3 ± 9.7		0.534	46.4 ± 0.9		0.077
Range	28.3–74.1			23.5–73.1		
<40 YO^1^	29 (25.0)	82.6		19 (22.1)	78.9	
≥40 YO	87 (75.0)	80.4		67 (77.9)	94.0	
Histology			0.765			0.207
IDC^2^	108 (93.1)			71 (82.6)	91.5	
Other	8 (6.9)			15 (17.4)	86.7	
Stage			<0.001			0.860
I	21 (18.1)	90.5		34 (39.5)	91.2	
IIA	48 (41.4)	93.8		46 (53.5)	89.1	
IIB	26 (22.4)	73.1		6 (7.0)	100.0	
IIIA	13 (11.2)	67.7		0 (0)	NA	
IIIB	0 (0)	NA		0 (0)	NA	
IIIC	8 (6.9)	25.0		0 (0)	NA	
Nuclear grade			0.503			0.134
1	0 (0)	NA		2 (2.3)	50.0	
2	31 (26.7)	80.3		16 (18.6)	87.5	
3	83 (71.6)	81.9		61(71.0)	93.4	
Unknown	2 (1.7)	50.0		7 (8.1)	85.7	
Histologic grade			0.898			0.395
1	1 (0.9)	100.0		2 (2.3)	100.0	
2	25 (21.5)	84.0		20 (23.3)	85.0	
3	87 (75.0)	79.1		56 (65.1)	94.6	
Unknown	3 (2.6)	66.7		8 (9.3)	75.0	
Adjuvant RTx^3^			0.052			0.814
Yes	79 (68.1)	77.1		51 (59.3)	92.2	
No	37 (31.9)	89.2		35 (40.7)	88.6	
NFKB1 (median: 25.60)			0.034			0.948
Low	62 (53.4)	74.2		39 (45.3)	92.3	
High	54 (46.6)	88.8		47 (54.7)	89.4	
NFKB2 (median: 297.30)			0.754			0.864
Low	55 (47.4)	81.7		46 (53.5)	89.1	
High	61 (52.6)	80.3		40 (46.5)	92.5	
RELA (median: 224.85)			0.023			0.832
Low	55 (47.4)	90.8		45 (52.3)	88.9	
High	61 (52.6)	72.1		41 (47.7)	92.7	
RELB (median: 61.25)			0.666			0.715
Low	57 (49.1)	82.3		44 (51.7)	90.9	
High	59 (50.9)	79.6		42 (48.8)	90.5	
REL (median: 65.48)			0.460			0.716
Low	67 (57.8)	77.6		34 (39.5)	91.2	
High	49 (42.2)	85.6		52 (60.5)	90.4	
TP53 (median: 221.06)			0.886			0.750
Low	59 (50.9)	79.7		42 (48.8)	92.9	
High	57 (49.1)	82.3		44 (51.2)	88.6	
FOXC1 (median: 126.94)			0.208			0.279
Low	59 (50.9)	86.3		42 (48.8)	88.1	
High	57 (49.1)	75.4		44 (51.2)	93.2	
TBP (median: 92.68)			0.609			0.158
Low	61 (52.6)	81.8		39 (45.3)	94.9	
High	55 (47.4)	80.0		47 (54.7)	87.2	
SP1 (median: 100.01)			0.001			0.193
Low	52 (44.8)	92.3		49 (57.0)	87.8	
High	64 (55.2)	71.6		37 (43.0)	94.6	
STAT3 (median:1599.68)			0.122			0.082
Low	58 (50.0)	86.1		43 (50.0)	86.0	
High	58 (50.0)	75.9		43 (50.0)	95.3	
IRF1 (median: 192.75)			0.695			0.599
Low	51 (44.0)	82.2		50 (58.1)	90.0	
High	65 (56.0)	80.0		36 (41.9)	91.7	

^1^Years old, ^2^Invasive ductal carcinoma, ^3^Radiotherapy.

**Table 3:** Impact of NFKB family mRNA expression levels on DRFS in patients with TNBC who received adjuvant doxorubicin chemotherapy (Cox regression).

Doxorubicin chemotherapy			
Clinical variables (N = 116)	HR	95% CI	*P*-value
Stage			<0.001
I	1.0	NA	
IIA	1.34	0.26–6.95	
IIB	3.60	0.75–17.36	
IIIA	7.07	1.36–36.91	
IIIC	20.19	4.00–101.87	
Adjuvant chemotherapy			0.664
(F)AC	1.0	NA	
AC-T	0.79	0.27–2.33	
NFKB1 (Median: 25.60)			0.527
Low	1.0	NA	
High	0.73	0.27–1.96	
RELA (Median: 224.85)			0.823
Low	1.0	NA	
High	0.88	0.27–2.83	
SP1 (Median: 100.01)			0.004
Low	1.0	NA	
High	4.97	1.68–14.73	
