# Supplementary material for: The relationship between nuclear factor (NF)-κB family gene expression and prognosis in triple-negative breast cancer (TNBC) patients receiving adjuvant doxorubicin treatment
Source: Sci Rep. 2016 Aug 22;6:31804. doi: 10.1038/srep31804 (PMC4992884; doi:10.1038/srep31804)

The relationship between Nuclear factor (NF)-κB family gene expression and prognosis in triple-negative breast cancer(TNBC) patients receiving adjuvant doxorubicin treatment

Ji-Yeon Kim[1,*], Hae Hyun Jung[5,*], Soomin Ahn[3], SooYoun Bae[4], Se Kyung Lee[4], Seok Won Kim[4], Jeong Eon Lee[4], Seok Jin Nam[4], Jin Seok Ahn[1], Young-Hyuck Im[1, 2] and Yeon Hee Park[1, 2,5]

[1]Division of Hematology-Oncology, Department of Medicine, [2]Biomedical Research Institute, [3]Cancer of Companion Diagnostics, [4]Department of Surgery, Samsung Medical Center, Seoul 06351, Korea; [5]Department of Health Sciences and Technology, SAIHST, Sungkyunkwan University, Seoul 06351, Korea

Supplementary Figure Legends

Supplementary Fig. 1. Patient cohort (N=203)

Supplementary Fig. 2. Heatmap for NF-κB pathway associated gene expression

(A) All patients (N=203)

(B) Patients with adjuvant doxorubicin chemotherapy (N=116)

(C) Patients with adjuvant CMF chemotherapy (N=86)

Supplementary Fig. 3. Relationship among NF-κB pathway-associated gene expression in TNBC with adjuvant doxorubicin chemotherapy

Supplementary Table 1. Relationships among expression of NFKB pathway-associated genes in TNBC

Supplementary Table 1 Relationships among expression of NFKB pathway-associated genes in TNBC

**Clinical variables**	**NFKB1**	**NFKB2**	**RELA**	**RELB**	**REL**	**TP53**	**FOXC1**	**TBP**	**SP1**	**STAT3**	**IRF1**
**NFKB1**											
Pearson R	1	-0.074	-0.385	0.084	0.694	0.041	-0.366	0.051	-0.491	-0.286	-0.241
P-value		0.429	<0.001	0.370	<0.001	0.665	<0.001	0.587	<0.001	0.002	0.009
**NFKB2**											
Pearson R	-0.074	1	0.825	0.728	-0.114	-0.067	0.230	0.541	0.599	0.535	0.391
P-value	0.429		<0.001	<0.001	0.225	0.477	0.013	<0.001	<0.001	<0.001	<0.001
**RELA**											
Pearson R	-0.385	0.825	1	0.560	-0.441	-0.033	0.449	0.430	0.845	0.697	0.453
P-value	<0.001	<0.001		<0.001	<0.001	0.728	<0.001	<0.001	<0.001	<0.001	<0.001
**RELB**											
Pearson R	0.084	0.728	0.560	1	0.027	0.203	0.127	0.391	0.349	0.549	0.612
P-value	0.370	<0.001	<0.001		0.777	0.029	0.176	<0.001	<0.001	<0.001	<0.001
**REL**											
Pearson R	0.694	-0.114	-0.441	0.027	1	0.033	-0.367	0.082	-0.542	-0.313	-0.222
P-value	<0.001	0.225	<0.001	0.777		0.727	<0.001	0.382	<0.001	0.001	0.017
**TP53**											
Pearson R	0.041	-0.067	-0.033	0.203	0.033	1	-0.009	-0.050	-0.079	-0.012	0.081
P-value	0.665	0.477	0.728	0.029	0.727		0.925	0.595	0.399	0.900	0.385
**FOXC1**											
Pearson R	-0.366	0.230	0.449	0.127	-0.367	-0.009	1	0.007	0.455	0.273	0.193
P-value	<0.001	0.013	<0.001	0.176	<0.001	0.925		0.942	<0.001	0.003	0.038
**TBP**											
Pearson R	0.051	0.541	0.430	0.391	0.082	-0.050	0.007	1	0.324	0.249	0.158
P-value	0.587	<0.001	<0.001	<0.001	0.382	0.595	0.942		<0.001	0.007	0.090
**SP1**											
Pearson R	-0.491	0.599	0.845	0.349	-0.542	-0.079	0.455	0.324	1	0.619	0.398
P-value	<0.001	<0.001	<0.001	<0.001	<0.001	0.399	<0.001	<0.001		<0.001	<0.001
**STAT3**											
Pearson R	-0.286	0.535	0.697	0.549	-0.313	-0.012	0.273	0.249	0.619	1	0.643
P-value	0.002	<0.001	<0.001	<0.001	0.001	0.900	0.003	0.007	<0.001		<0.001
**IRF1**											
Pearson R	-0.241	0.391	0.453	0.612	-0.222	0.081	0.193	0.158	0.398	0.643	1
P-value	0.009	<0.001	<0.001	<0.001	0.017	0.385	0.038	0.090	<0.001	<0.001	

Supplementary Fig. 1.

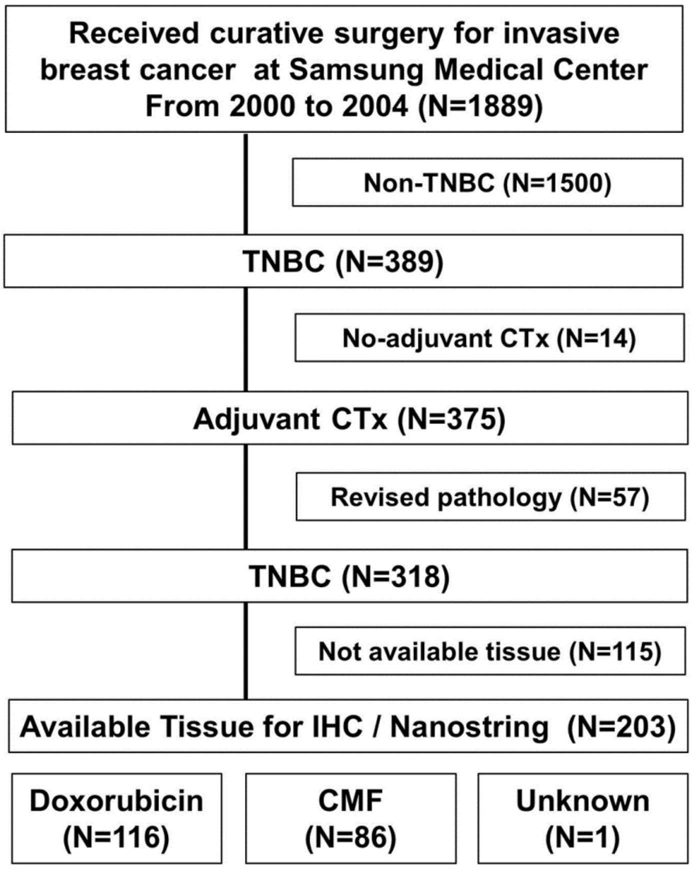

Supplementary Fig. 2.

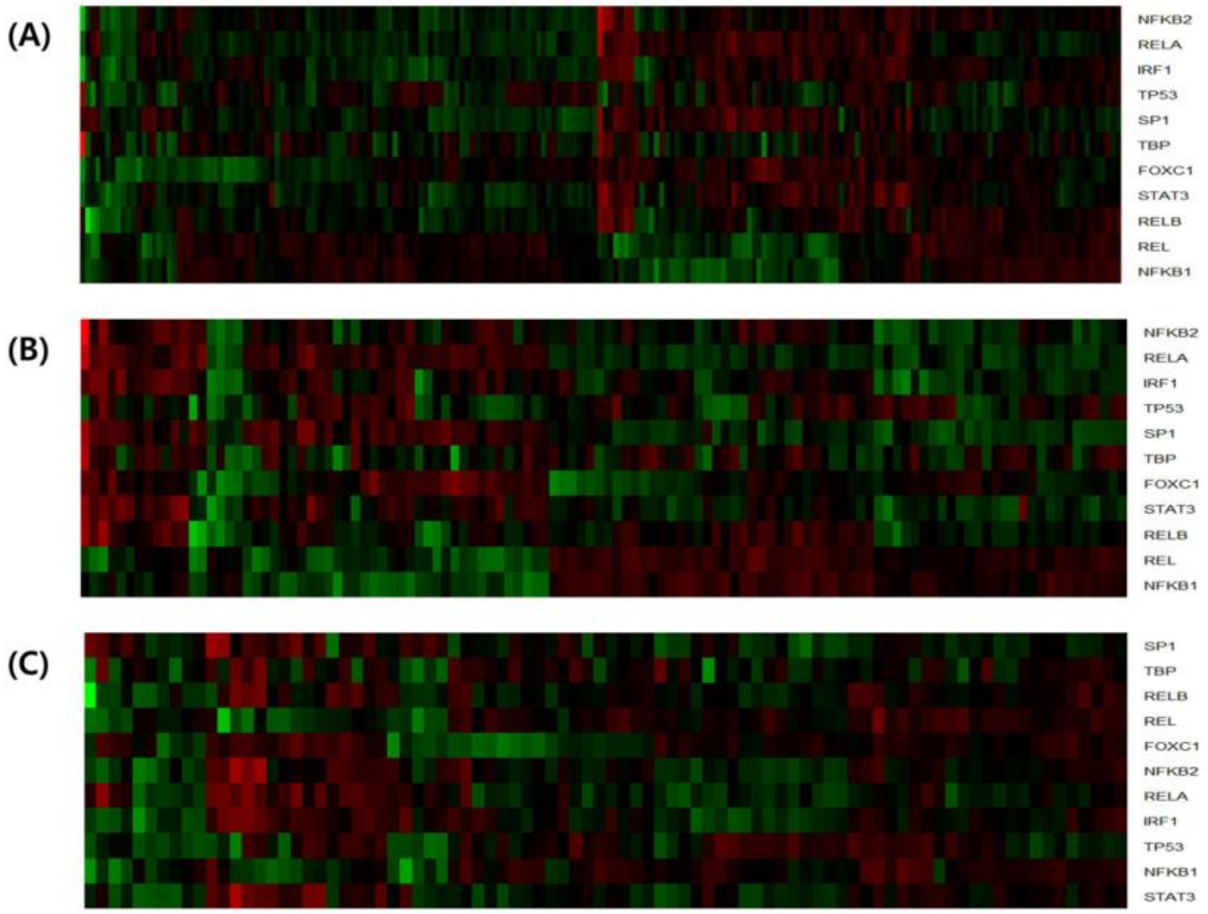

Supplementary Fig. 3

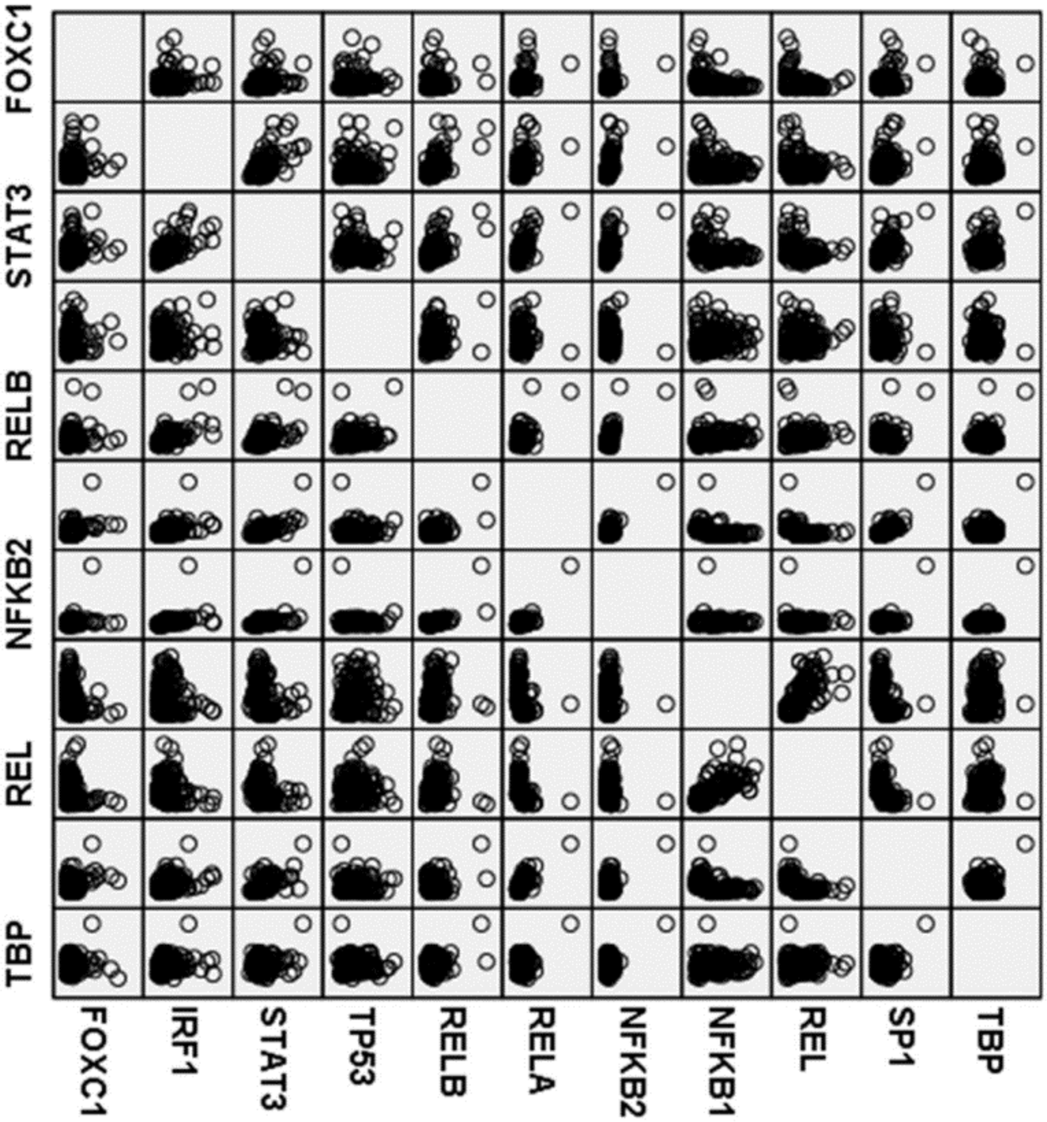

Supplement: Supplementary Information [file srep31804-s1.pdf]
